# Biased Language in Simulated Handoffs and Clinician Recall and Attitudes

**DOI:** 10.1001/jamanetworkopen.2024.50172

**Published:** 2024-12-17

**Authors:** Austin Wesevich, Erica Langan, Ilona Fridman, Sonya Patel-Nguyen, Monica E. Peek, Victoria Parente

**Affiliations:** 1 Section of Hematology/Oncology, Department of Medicine, The University of Chicago, Chicago, Illinois; 2 Duke University, Durham, North Carolina; 3 Center for Discovery and Innovation, Hackensack Meridian Health, Hackensack, New Jersey; 4 Lombardi Comprehensive Cancer Center, Georgetown University, Washington, DC; 5 Division of Hospital Medicine, Department of Medicine, Duke University, Durham, North Carolina; 6 Division of Hospital Medicine, Department of Pediatrics, Duke University, Durham, North Carolina; 7 Section of General Internal Medicine, Department of Medicine, The University of Chicago, Chicago, Illinois

## Abstract

**Importance:**

Poor-quality handoffs can lead to medical errors when transitioning patient care. Biased language within handoffs may contribute to errors and lead to disparities in health care delivery.

**Objective:**

To compare clinical information recall accuracy and attitudes toward patients among trainees in paired cases of biased vs neutral language in simulated handoffs.

**Design, Setting, and Participants:**

Surveys administered from April 29 to June 15 and from July 20 to October 10, 2023, included 3 simulated verbal handoffs, randomized between biased and neutral, and measured clinical information recall, attitudes toward patients, and key takeaways after each handoff. Participants included residents in internal medicine, pediatrics, and internal medicine–pediatrics and senior medical students at 2 academic medical centers in different geographic regions of the US. Data were analyzed from November 2023 to June 2024.

**Exposures:**

Each participant received 3 handoffs that were based on real handoffs about Black patients at 1 academic center. These handoffs were each randomized to either a biased or neutral version. Biased handoffs had 1 of 3 types of bias: stereotype, blame, or doubt. The order of handoff presentation was also randomized. Internal medicine and pediatrics residents received slightly different surveys, tailored for their specialty. Internal medicine–pediatrics residents received the pediatric survey. Medical students were randomly assigned the survey type.

**Main Outcomes and Measures:**

Each handoff was followed by a clinical information recall question, an adapted version of the Provider Attitudes Toward Sickle Cell Patients Scale (PASS), and 3 free-response takeaways.

**Results:**

Of 748 trainees contacted, 169 participants (142 residents and 27 medical students) completed the survey (23% overall response rate), distributed across institutions, residency programs, and years of training (95 female [56%]; mean [SD] age, 28.6 [2.3] years). Participants who received handoffs with blame-based bias had less accurate information recall than those who received neutral handoffs (77% vs 93%; *P* = .005). Those who reported bias as a key takeaway of the handoff had lower clinical information recall accuracy than those who did not (85% vs 93%; *P* = .01). Participants had less positive attitudes toward patients per PASS scores after receiving biased compared with neutral handoffs (mean scores, 22.9 [3.3] vs 25.2 [2.7]; *P* < .001). More positive attitudes toward patients were associated with higher clinical information recall accuracy (odds ratio, 1.12; 95% CI, 1.02-1.22).

**Conclusions and Relevance:**

In this survey study of residents and medical students, biased handoffs impeded accurate transfer of key clinical information and decreased empathy, potentially endangering patients and worsening health disparities. Handoff standardization is critical to addressing racial bias and improving patient safety.

## Key Points

**Question:**

What is the impact of biased language in simulated verbal handoffs on recipient clinical information recall and attitude toward patients?

**Findings:**

In this survey study of 169 residents and medical students, participants had less accurate clinical information recall and less positive attitudes toward patients after hearing biased simulated verbal handoffs than after hearing neutral handoffs. Positive attitudes toward patients were associated with clinical information recall accuracy.

**Meaning:**

These results further support standardization of handoffs as called for by multiple organizations, critical to reducing biased language that can negatively impact clinicians’ perceptions of patients and reduce retention of key clinical information needed for patient care.

## Teaser

This survey study assesses the association of biased language during simulated inpatient handoffs with the recall accuracy of clinical information and clinician attitudes about patients among residents and medical students.

## Introduction

Clinician bias is one of many contributors to racial inequities in health care and health outcomes in the US, including in hospital settings.^1,2,3,4,5,6,7,8,9,10,11,12,13,14,15,16,17,18^ Clinician bias toward patients based on social identities and physical characteristics (eg, race and ethnicity) may affect clinician attitudes and beliefs about patients as well as impact clinical decisions.^19,20,21,22^ In a landmark study,^19^ physicians had more than twice the odds of recommending cardiac catheterization for White patients with chest pain in video clinical vignettes compared with Black patients. In a survey study,^21^ pediatricians with implicit pro-White bias were less likely to prescribe postoperative opioids to Black patients than to White patients.

Studies of electronic medical records^23,24^ suggest that clinician notes about Black patients have higher odds of including negative descriptors (eg, aggressive) and disbelieving patient narratives (ie, doubt) compared with those about White patients. Clinicians have also been shown to use biased language more often during transfers of care when verbally describing Black patients compared with those of other races.^25^ Bias in physician communication, whether written or verbal, is particularly harmful because bias can be transmitted between clinicians.^26^ In a clinical vignette study,^26^ exposure to biased language in a hypothetical patient’s medical note was associated with more negative attitudes toward the patient and less aggressive pain management.

Clinicians communicate with each other about hospitalized patients through notes in the electronic medical record, transfers of care (ie, patient handoffs), and other informal processes. The hospital setting is a particularly vulnerable time for patients—continuity of care is often broken, and clinicians have to make urgent decisions with limited information. These circumstances increase the risk of the use of implicit biases, which are cognitive shortcuts, and increase the risk of medical errors.^27,28,29^ Thus, accurate physician communication about patients in hospital settings may be one important strategy to reduce racial disparities, including medical errors, which disproportionately affect racially minoritized populations.^30,31,32,33^

We explored the impact of biased language during inpatient handoffs on the recall accuracy of clinical information and on clinician attitudes about the patients for whom they are assuming responsibility. Handoffs typically entail clinicians communicating brief descriptions of patients and any outstanding medical issues or anticipatory guidance for oncoming clinicians when transferring patients’ care.^34,35,36^ Communication failures have been identified as the root cause of more than 60% of the sentinel events reported to the Joint Commission.^37^ There is an increased risk of medical errors with inadequate handoffs,^38,39^ which has led to requirements for standardizing the handoff process, particularly among house staff.^34,35,40^

## Methods

### Study Design and Setting

This randomized survey study measured recall accuracy of clinical information and clinician attitudes toward patients among internal medicine, pediatrics, and internal medicine–pediatrics residents as well as medical students who had completed required clerkships at 2 academic medical centers in geographically distinct areas of the US. Residents were surveyed from April 29 to June 15, 2023, and senior medical students from July 20 to October 10, 2023. This study was approved by the Duke University Health System Institutional Review Board as an extension of prior work that required audio recording and transcription of actual patient handoffs at Duke University.^25^ This survey study was determined to be exempt by The University of Chicago Institutional Review Board because the identity of respondents cannot be readily determined. Reporting guidelines for survey studies by the American Association for Public Opinion Research (AAPOR) were followed.

### Study Procedures

Residents and senior medical students were invited via email to participate. Nonrespondents received up to 4 reminder emails. Internal medicine residents received internal medicine surveys (eAppendix 1 in Supplement 1) while pediatrics and internal medicine–pediatrics residents received pediatric surveys (eAppendix 2 in Supplement 1). Medical students were randomized to either internal medicine or pediatric surveys. Residents were offered a $25 electronic gift card after survey completion, while medical students were eligible for a raffle of five $100 electronic gift cards.^26^

### Survey Design

Two surveys were developed consisting of either 3 internal medicine or 3 pediatric handoffs. The simulated verbal handoffs in both surveys were based on real biased handoffs about Black patients at 1 academic center from a prior study conducted by our group.^25^ Each of the handoffs selected from the prior study had a different type of bias present: stereotype, blame, or doubt. While there are many ways to classify bias and its interference with processing information,^41,42,43^ these categories of bias were selected for the prior study^25^ based on literature evaluating stigmatizing language in written communication.^24,26,44,45,46,47^ Thus, there were 3 internal medicine handoffs, each with a different type of bias, and 3 pediatric handoffs with the same 3 types of bias. Each of these biased handoffs referred to a different clinical scenario; 6 different patients, 3 adult and 3 pediatric, were presented. Slight changes were made to the original biased handoffs to protect patient confidentiality.

The study team then developed 6 neutral-language versions of these biased handoffs. For example, the blame-based bias phrasing of an adult patient with diabetes who “cut her dose in half 2 months ago because she was having some falls and thought she was getting hypoglycemic but never actually checked her blood glucose” was rephrased to describe someone who “cut her dose in half after experiencing hypoglycemic symptoms” (Table 1). Audio files were recorded for both biased and neutral versions to serve as simulated verbal handoffs. The 3 handoffs within a given survey were each randomized to either biased or neutral versions of each patient handoff, and the order of those 3 handoffs was also randomized. This produced a total of 48 versions of the internal medicine survey and 48 versions of the pediatric survey.

**Table 1.** Survey Tool Summary by Handoff

Handoff	Bias	Patient age/sex	Biased phrasing	Changes for neutral phrasing	Recall question (correct answer)
**Internal Medicine handoffs**					
Urinary tract infection and altered mental status					
	Stereotype	74 y/Female	“74-Year-old lady came in altered with pyuria.”	“74-Year-old female with pyuria and altered mental status”	Overnight fever plan if hemodynamically stable? (Repeat blood and urine cultures)
			“Her baseline now is A&O × 3 but a little bit irritable and like, skeptical. Like grouchy I guess is a good word for her. So just like, try not to go in that room if you don’t have to.”	“…her baseline of A&O × 3. She has had a difficult admission with her changes in mental status.”	
			“She’ll probably, *hopefully (!)* go tomorrow.”	“She’ll probably go tomorrow.”	
Ovarian cancer and diabetes					
	Blame	72 y/Female	“She had been diagnosed with ovarian cancer about 2 years ago and at that time was like, ‘Well, I don’t have to worry about my sugars anymore.’ And really stopped checking them.”	“She’s been more focused on her ovarian cancer and stopped checking her glucose.”	What to do if glucose goes below 300? (Allow PO intake)
			“Cut her dose in half 2 months ago because she was having some falls and thought she was getting hypoglycemic but never actually checked her blood glucose.”	“Cut her dose in half after experiencing hypoglycemic symptoms.”	
			“You can let her eat if she’s bugging you.”	“You can remove her NPO status and order her a diet.”	
Prostate cancer and bunions					
	Doubt	62 y/Male	“He is complaining of 10 out of 10 pain at like his bunions…. I told the nurse …not to page you about this. Because it’s like, if he’s looking away and you tap on it, it doesn’t hurt. He’s already got Tylenol and a lidocaine patch on it. Literally, don’t do anything for that. Yeah, ha. It’s a bunion. It’s not gout.”	“He has bilateral bunions that are causing 10 out of 10 pain. I don’t expect bunions to be that painful, and it doesn’t look like gout. For now, I ordered Tylenol and a lidocaine patch for the pain and to see if that helps.”	Which lab value to follow up and act upon overnight? (Potassium)
**Pediatric handoffs**					
Seizure evaluation					
	Stereotype	6 mo/Male	“Ex-23-weeker.”	“Born at 23 weeks.”	Description of spell or seizurelike activity at home? (Head shaking and tremor)
			“Mom does seem a little bit frustrated about being in the hospital…literally screamed at me because she thought I was misgendering her baby when I was instead referring to the nurse. Mom’s a little excitable.”	“Mom is disappointed to be back in the hospital so soon after his discharge from the NICU.”	
			“I talked to her at length. Made sure I got all his feeds and all his meds exactly like she wanted them. So, hopefully you won’t have a lot of discussion overnight. …And so, I apologize in advance if you get like a ‘We want to leave right now.’…We both talked to her about the necessity for the admission…She just was in general frustrated.”	“…[Mom] is wondering about the need to be back inpatient. I spent a while talking to her about the importance of capturing an event on EEG to know if these are seizures. I also went over his home meds and feeds with her to make sure they were correct and to avoid any errors.”	
Developmental delay and failure to thrive					
	Blame	13 mo/Female	“We were told that mom was like hypo-diluting formula—but I guess when they told her that maybe she decided to interpret that as, ‘Oh, that’s wrong. I just won’t use formula then?’ So, it could be that she’s just not getting enough…in to meet her nutritional demand.”	“It also sounds like she may not be getting enough nutritionally to meet her demands as there were some miscommunications about her formula mixing.”	What diet ordered in the hospital? (Toddler diet)
			“She takes solid food, maybe half a handful a day, and some rice cereal diluted in almond milk. Mom also explicitly said like, ‘I’m afraid of overfeeding my baby.’ Which is shocking, given like she’s first percentile growth. …There’s definitely something going on with Mom.”	“She is no longer on formula, but gets rice cereal mixed with almond milk and a small amount of solids.”	
Optic neuritis and anxiety					
	Doubt	14 y/Male	“Assess whether there’s concern for anaphylaxis vs not. Part of the reason why I have a concern for possibly not is he, at baseline, I don’t know if you have met him, is a pretty darn anxious kid.”	“Assess for anaphylaxis.”	What intern had already done for chest tightness? (Slowed down infusion rate)

Abbreviations: A & O × 3, alert and oriented to person, place, and time; EEG, electroencephalography; NICU, neonatal intensive care unit; NPO, nothing by mouth; PO, by mouth.

### Predictive Variable

The 3 handoffs in a survey were each randomized to being biased or neutral. Thus, the internal medicine survey contained handoffs about (1) a woman with altered mental status, which either had stereotype-based bias or was neutral; (2) a woman with diabetes, which either had blame-based bias or was neutral; and (3) a man with bunions, which either had doubt-based bias or was neutral. The pediatric survey was similarly structured (Table 1).

### Outcome Variables

Clinical information recall accuracy was assessed after each handoff through a multiple-choice question about key clinical information; the same question was asked for both biased- and neutral-language versions. Responses were dichotomized as correct vs incorrect.

Clinician attitudes toward patients was assessed using adapted versions of the Provider Attitudes Toward Sickle Cell Patients Scale (PASS), a validated survey assessing clinician attitudes about patients with sickle cell disease that has been used in other patient populations.^48,49,50^ PASS item responses were scored from 1 to 5, with higher scores indicating more positive attitudes toward patients.^26,48^ We included 9 items in the internal medicine survey (PASS-9) but omitted the substance abuse item for the pediatric survey (PASS-8). PASS responses were totaled across items, yielding PASS-9 total scores ranging from 9 to 45 and PASS-8 total scores ranging from 8 to 40.

Key takeaways were assessed through participants’ 3 free-response takeaways after each handoff. Open-ended takeaways were close coded as clinical information, bias, and/or other. Bias takeaway referred to biased phrases or subjective comments about a patient being a key takeaway, such as describing the woman with altered mental status as grouchy. Clinical information takeaway referred to relevant information from the handoff necessary to answer the key clinical information multiple choice question. Because takeaway messages not focused on either bias or clinical information (eg, including “foot pain” for the patient with bunions but not describing key management recommendations) were less important for this analysis, they were compiled into the category of other. Since 3 takeaways could be listed, participants could have both bias and clinical information takeaways for a given handoff.

### Covariates

Participants self-reported their sociodemographic and educational characteristics (ie, age, gender, race and ethnicity, type of training program, and level of experience). Gender categories were “female,” “male,” “nonbinary,” “other,” and “prefer not to answer”; there were no responses of “nonbinary” or “other. Racial and ethnic categories of American Indian or Alaska Native, Black or African American, East Asian (eg, Chinese, Korean, Japanese), Hispanic or Latin American, Middle Eastern or North African, Native Hawaiian or Other Pacific Islander, South Asian (eg, Indian, Pakistani), Southeast Asian (eg, Cambodian, Filipino, Vietnamese, Hmong), White, or other were recommended by expert opinion.^51^ Participants could select multiple racial and ethnic categories. Race was compared as White vs non-White participants given insufficient numbers to compare Black vs non-Black participants. Ethnicity was dichotomized into Latinx vs non-Latinx. Training program was dichotomized into preliminary vs categorical. Level of experience was ordinal on a postgraduate year scale, with medical students considered postgraduate year 0.

### Statistical Analysis

We used descriptive statistics for survey response rates and participant sociodemographic and education characteristics. We assessed the impact of bias outcomes of interest. First, we compared clinical information recall accuracy between biased- and neutral-language handoffs using χ^2^ tests. Second, we compared clinician attitudes toward patients (item and total PASS scores) between biased- and neutral-language handoffs using Wilcoxon rank sum tests. Third, we compared both clinical information recall accuracy and clinician attitudes toward patients (item and total PASS scores) between study participants with and without a bias takeaway using χ^2^ and Wilcoxon rank sum tests, respectively. Fourth, we compared the presence of a bias takeaway between those with and without a clinical information takeaway using χ^2^ tests. We also compared clinical information recall accuracy between participants with and without a clinical information takeaway to assess takeaway close coding. Fifth, we explored associations between reporting a bias takeaway and participant sociodemographic and educational factors using χ^2^ tests and logistic regression models. Finally, we compared both clinical information recall accuracy and clinician attitudes towards patients by the presence of a bias takeaway using multilevel logistic regression models. Analyses were performed when combining all handoff responses as well as when stratifying by handoff type (stereotype vs blame vs doubt) and survey type (internal medicine vs pediatric).

Additionally, we were interested in the association between clinician attitudes toward patients and their clinical information recall accuracy. We first explored this through unadjusted multilevel logistic regression models. To account for 3 handoffs per survey, we clustered by participant. To adjust for important participant- and handoff-level covariates, we also used multivariable multilevel logistic regression models clustered by participant. The multivariable models were developed using stepwise backward elimination to systematically remove covariates with the largest *P* value until all remaining variables were statistically significant or clinically relevant. Sociodemographic and educational covariates as well as handoff fixed and random effects (ie, which handoff and duration and order of handoffs) were not statistically significant in multivariable models. The final model included participant race, handoff bias randomization, order of handoffs, duration of handoffs, and bias takeaway because of their relevance to the study topic, as well as a clinical information takeaway because of statistical significance. A 2-sided threshold of *P* < .05 indicated statistical significance. All analyses were performed from November 2023 to June 2024 using Stata, version 16.1 (StataCorp LLC).

## Results

Of 433 residents contacted, a total of 142 (33%) responded, and of 315 senior medical students contacted, 27 (9%) responded to the survey, yielding an overall study population of 169 participants (23% response rate). This comprised responses to 499 simulated handoffs. Study participants had a mean (SD) age of 28.6 (2.3) years; 95 (56%) were female, 69 (41%) were male, and 5 (3%) preferred not to answer. In terms of race and ethnicity, 3 (2%) were Black or African American; 15 (9%), East Asian; 11 (7%), Hispanic or Latin American; 6 (4%), Middle Eastern or North African; 29 (17%), South Asian; 3 (2%), Southeast Asian; 102 (60%), White; and 14 (8%), not reported. Participants were well distributed with 80 (47%) from internal medicine residency programs and 51 (30%) from pediatrics or internal medicine–pediatrics programs, with overall participation roughly evenly split between the 2 participating institutions, and 58 (34%) in their intern year of residency (Table 2). Seven study participants (4%) did not complete all 3 simulated handoffs; partial responses were included.

**Table 2.** Participant Sociodemographic and Educational Characteristics

Characteristic	Frequency, No. (%)
Program	
Internal medicine	80 (47)
Internal medicine–pediatrics	17 (10)
Pediatrics	34 (20)
Other residency	11 (7)
Medical school	27 (16)
Institution	
A	88 (52)
B	81 (48)
Postgraduate year	
0 (medical school)	27 (16)
1	58 (34)
2	31 (18)
3	47 (28)
4	6 (4)
Age, mean (SD), y	28.6 (2.3)
Gender	
Female	95 (56)
Male	69 (41)
Nonbinary	0
Other	0
Prefer not to answer or blank	5 (3)
Race and ethnicity^a^	
American Indian or Alaska Native	0
Black or African American	3 (2)
East Asian (eg, Chinese, Korean, Japanese)	15 (9)
Hispanic or Latin American	11 (7)
Middle Eastern or North African	6 (4)
South Asian (eg, Indian, Pakistani)	29 (17)
Southeast Asian (eg, Cambodian, Filipino, Vietnamese, Hmong)	3 (2)
White	102 (60)
Prefer not to answer or blank	14 (8)

^a^ Participants could check multiple boxes for race and ethnicity, so total sums to more than 100%.

### Clinical Information Recall Accuracy

Across all handoffs, clinical information recall accuracy was lower after receiving biased handoffs than neutral-language handoffs, although the difference was not statistically significant (88% vs 92%; *P* = .12) (Table 3). However, when participants received handoffs with blame-based bias, they had significantly lower clinical information recall accuracy than when they received neutral-language handoffs (77% vs 93%; *P* = .005).

**Table 3.** Clinical Information Recall Accuracy and Clinician Attitudes Toward Patients Compared by Handoff Bias Randomization and by Bias Takeaway

Handoff bias type	Biased handoff	Neutral handoff	*P* value	Bias takeaway	No bias takeaway	*P* value
Clinical information recall accuracy, %						
Stereotype	89	91	.71	85	95	.04
Blame	77	93	.005	77	90	.02
Doubt	98	93	.16	100	94	.20
Overall	88	92	.12	85	93	.01
Internal medicine clinician attitudes toward patients, mean (SD) PASS-9 score^a^						
Stereotype	24.9 (3.4)	29.0 (3.3)	<.001	24.8 (3.0)	27.6 (4.0)	<.001
Blame	26.9 (3.5)	28.5 (3.3)	.01	27.7 (4.1)	27.7 (3.1)	.90
Doubt	25.9 (3.4)	28.2 (3.7)	<.001	25.1 (4.6)	27.6 (3.2)	.001
Overall	25.9 (3.5)	28.5 (3.4)	<.001	26.1 (4.1)	27.6 (3.4)	<.001
Pediatric clinician attitudes toward patients, mean (SD) PASS-8 score^b^						
Stereotype	21.0 (3.5)	25.1 (2.3)	<.001	22.1 (3.9)	24.2 (2.7)	.01
Blame	23.5 (3.2)	25.1 (2.3)	.02	22.7 (2.8)	25.0 (2.7)	.008
Doubt	25.3 (1.9)	25.4 (2.0)	.90	25.5 (2.2)	25.3 (1.9)	.53
Overall	23.1 (3.5)	25.2 (2.2)	<.001	23.0 (3.6)	24.9 (2.4)	<.001

Abbreviation: PASS: Provider Attitudes Toward Sickle Cell Patients Scale.

^a^ Scores range from 9 to 45, with higher scores indicating a more positive attitude toward a patient.

^b^ Scores range from 8 to 40, with higher scores indicating a more positive attitude toward a patient.

### Clinician Attitudes Toward Patients

Total PASS-8 scores were significantly lower, indicating less positive attitudes, after biased handoffs compared with neutral-language handoffs (22.9 [3.3] vs 25.2 [2.7]; *P* < .001) (Table 4). Each PASS item was significantly lower for biased handoffs compared with neutral handoffs (Table 4). For example, participants had lower empathy scores (PASS item 2) when hearing biased handoffs than neutral handoffs (3.1 [0.6] vs 3.3 [0.5]; *P* < .001). In addition, each of the handoffs had lower total PASS scores for biased-language (vs neutral-language) versions (eg, PASS-9 score of 26.9 [3.5] vs 28.5 [3.3] for the adult patient with diabetes with or without blame-based bias; *P* = .01) except for the pediatric doubt-based bias handoff, where no difference was found (Table 3).

**Table 4.** Clinician Attitudes (PASS Score) by Handoff Bias Type and by Bias Takeaway

PASS item^a^	PASS score		*P* value	PASS score		*P* value
	Biased	Neutral		Bias takeaway	No bias takeaway	
1. How much you like patient and/or family	2.7 (0.6)	3.0 (0.4)	<.001	2.6 (0.7)	3.0 (0.5)	<.001
2. How much empathy for patient and/or family	3.1 (0.6)	3.3 (0.5)	<.001	3.1 (0.7)	3.2 (0.5)	.30
3. How much respect for patient and/or family	2.9 (0.5)	3.1 (0.4)	<.001	2.9 (0.5)	3.1 (0.4)	<.001
4. Patient and/or family frustrating to take care of	2.8 (0.9)	3.3 (0.8)	<.001	2.8 (0.9)	3.2 (0.8)	<.001
5. Feel glad went into medicine	3.1 (0.6)	3.2 (0.6)	.003	3.0 (0.7)	3.2 (0.5)	<.001
6. Overreport (exaggerate) discomfort	2.7 (0.7)	3.0 (0.6)	<.001	2.8 (0.7)	2.9 (0.6)	.01
7. Fail to comply with medical advice	2.7 (0.7)	3.0 (0.6)	<.001	2.7 (0.8)	2.9 (0.6)	<.001
8. Try to manipulate you or other providers	2.9 (0.6)	3.2 (0.6)	<.001	2.9 (0.7)	3.1 (0.6)	<.001
9. Abuse drugs and/or alcohol	3.2 (0.6)	3.3 (0.7)	.05	3.3 (0.7)	3.3 (0.6)	.75
Overall PASS-8^b^	22.9 (3.3)	25.2 (2.7)	<.001	22.9 (3.7)	24.6 (2.9)	<.001
Overall PASS-9^c^	25.9 (3.5)	28.5 (3.4)	<.001	26.1 (4.1)	27.6 (3.4)	<.001

Abbreviation: PASS, Provider Attitudes Toward Sickle Cell Patients Scale.

^a^ Items 4 and 6 to 9 were reverse coded. Item 9 was only asked on the internal medicine survey (PASS-9). Item responses were scored from 1 to 5, with higher scores indicating more positive attitudes toward patients.

^b^ Scores range from 8 to 40, with higher scores indicating a more positive attitude toward a patient.

^c^ Scores range from 9 to 45, with higher scores indicating a more positive attitude toward a patient.

Participants with higher PASS-8 scores had a statistically significant increased odds of clinical information recall accuracy (odds ratio [OR], 1.12; 95% CI, 1.02-1.22) (Table 5). Of the 9 individual PASS items, 4 met statistical significance for positive associations with clinical information recall accuracy (affinity, empathy, respect, and absence of frustration), with the strongest association for empathy (OR, 2.09; 95% CI, 1.23-3.54; *P* = .006). In multivariable multilevel models, empathy remained positively associated with clinical information recall accuracy after adjusting for participant race, handoff bias randomization, bias takeaway, and clinical information takeaway (adjusted OR, 1.95; 95% CI, 1.12-3.41; *P* = .02).

**Table 5.** Clinician Attitude (PASS Items) Associated With Clinical Information Recall Accuracy in Unadjusted and Adjusted Multilevel Models

PASS item^a^	Unadjusted models		Adjusted models^b^	
	OR (95% CI)	*P* value	AOR (95% CI)	*P* value
1. How much you like patient and/or family	1.93 (1.16-3.19)	.01	1.76 (0.99-3.12)	.05
2. How much empathy for patient and/or family	2.09 (1.23-3.54)	.006	1.95 (1.12-3.41)	.02
3. How much respect for patient and/or family	1.89 (1.01-3.54)	.05	1.53 (0.77-3.01)	.22
4. Patient and/or family frustrating to take care of	1.55 (1.08-2.23)	.02	1.46 (0.98-2.19)	.06
5. Feel glad went into medicine	1.48 (0.91-2.43)	.12	1.36 (0.82-2.25)	.23
6. Overreport (exaggerate) discomfort	1.25 (0.78-1.98)	.35	1.30 (0.79-2.15)	.30
7. Fail to comply with medical advice	0.99 (0.63-1.55)	.96	0.90 (0.58-1.41)	.66
8. Try to manipulate you or other providers	1.42 (0.86-2.36)	.17	1.37 (0.81-2.31)	.24
9. Abuse drugs and/or alcohol	1.14 (0.59-2.21)	.70	1.41 (0.66-3.02)	.38
Overall PASS-8 score	1.12 (1.02-1.22)	.01	1.11 (1.00-1.22)	.05
Overall PASS-9 score	1.15 (1.03-1.29)	.02	1.16 (1.02-1.33)	.02

Abbreviations: AOR, adjusted odds ratio (OR); PASS, Provider Attitudes Toward Sickle Cell Patients Scale.

^a^ PASS-8 for pediatric and internal medicine surveys includes items 1 to 8; PASS-9 for internal medicine surveys includes item 9. Higher scores indicate a more positive attitude toward a patient.

^b^ Multilevel logistic regression models adjusted for participant race, handoff bias randomization, order of handoffs, duration of handoffs, having a bias takeaway, and having a clinical information takeaway.

### Key Takeaways

#### Bias Takeaway

After receiving biased handoffs, participants had a bias takeaway 53% of the time compared to 14% of the time after neutral handoffs. The frequency of bias takeaways did not differ by participant sociodemographic or educational characteristics except for a higher frequency among those receiving pediatric handoffs than internal medicine handoffs (40% vs 31%; *P* = .02). Clinical information recall accuracy was lower for those with a bias takeaway than those without (85% vs 93%; OR, 0.47; 95% CI, 0.26-0.85; *P* = .01) (Table 3). Clinician attitudes toward patients were less positive when they had a bias takeaway than when they did not (OR, 0.31; 95% CI, 0.21-0.46; *P* < .001).

#### Clinical Information Takeaway

Participants mentioned clinical information from the handoff relevant to the information recall question as a key takeaway 34% of the time. Clinical information recall accuracy was higher for those with a clinical information takeaway than those without (95% vs 88%; *P* = .01). Clinical information takeaways were less frequent for biased (vs neutral) handoffs (31% vs 37%; *P* = .18), as well as when bias takeaways were present than when they were not (29% vs 37%; *P* = .09), though these were not statistically significant differences.

## Discussion

To our knowledge, this survey study is the first to quantify the potential impact of bias created by stigmatizing language during verbal handoffs with clinical information recall accuracy and attitudes toward patients. Our data suggest that when residents and medical students hear handoffs about patients that contain blame-based biased language vs neutral language, they recall clinical information less accurately and that when they hear handoffs with any type of bias vs neutral-language handoffs, they have less empathy for patients. Because the biased language used in this study was derived from actual handoffs about Black patients, and multiple studies have demonstrated greater use of stigmatizing language when describing Black patients,^23,24,25^ our study suggests that racial bias exhibited during verbal patient handoffs can adversely impact quality of care for Black patients. Clinical information recall accuracy and positive patient attitudes were particularly diminished when bias was reported as a key takeaway from the verbal handoff. Our study suggests that biased language can be an important barrier to safe and effective handoffs for racially and ethnically minoritized patients.

Our study has implications for health care disparities in clinical settings. Prior research^34,35,38,39,40,52^ indicates that poor handoffs increase the risk of medical errors and are associated with worse clinical outcomes. One study^53^ found that cross-coverage was associated with a 6-fold higher odds of preventable adverse events among house staff, suggesting that improving handoffs may reduce medical errors. Another study^54^ found that poor handoffs and other communication errors were contributing factors in 35% of malpractice claims involving house staff. The simulated handoffs in our study were only slightly revised versions of real resident handoffs; thus, decreased clinical accuracy of handoffs is likely occurring in clinical settings because of racially biased language. Lower quality of handoffs may contribute to the disproportionate rate of medical errors among racially minoritized patients.^30,31,32,33^ In a review of marginalized hospital patients,^32^ communication errors were the most common problem that led to patient safety errors. Unbiased handoffs in our study were associated with more empathy, which in turn, has been associated with patient-centered care, an established measure of health care quality and a predictive factor associated with positive health outcomes.^55,56,57,58,59^

Thus, clinician handoffs represent a uniquely vulnerable time for patients where biased language can erode clinician empathy and decrease the effective transfer of clinical information, subsequently increasing the risk for medical errors and harm to racially minoritized patients. This may be particularly true for inpatient settings, where patients may be outside their medical home and separated from their medical records and primary clinical team. Sun and colleagues^23^ demonstrated that outpatient notes had fewer negative descriptors than inpatient notes, which they attributed to the continuity of care found in outpatient settings. Because handoffs often occur at the end of the day when clinicians are more likely to be fatigued, they may be more likely to use biased or negative language.^60,61,62^ The night shift, seen primarily in hospital medical settings, might be associated with increased fatigue and higher cognitive burden,^63,64^ circumstances that increase the use of cognitive shortcuts (eg, stereotypes, bias)^65,66,67^ and the risk of medical errors.^68,69^

It is important to note that this survey study measured the impact of biased language from real handoffs about Black patients on the recipient’s clinical information recall and attitudes toward patients. Thus, the outcomes indicate whether a degree of bias transfer occurred between clinicians. The biases that clinicians have may systematically perpetuate themselves in notes and handoffs since stereotype-consistent information is often preferentially maintained compared with stereotype-inconsistent information.^70,71^ Additionally, negative information is processed faster, leads to greater impression formation, and is more likely to be socially transmitted.^72,73^ This could lead to psychologically rewarding patterns of perpetuating negative information about patients, similar to a clinician game of “telephone.”

The Joint Commission’s National Patient Safety Goals require a standardized approach to handoff communication for all clinicians, with specific guidelines that handoffs include “accurate information.”^40^ Handoffs are ideally given in environments free of distractions, but our study demonstrated that racially biased language in the handoff can act as a distractor itself and decrease the accurate transfer of clinical information. The landmark I-PASS study demonstrated that implementing a standardized handoff reduced the relative incidence of preventable adverse events in teaching hospitals by 23%.^36^ For over a decade, the Accreditation Council for Graduate Medical Education has required that all residents achieve competence in handoff communication.^74,75^ In its policy statement about standardized handoffs for patients in the pediatric emergency department, the American Academy of Pediatrics noted the tendency for cognitive biases to affect clinician decision-making through mechanisms like framing effects, where decisions are influenced by the way scenarios are presented, and confirmation bias, where clinicians seek confirmatory data and disregard conflicting data.^35^ While this report called for family-centered care to improve health care, safety, and patient satisfaction, it did not call for training to address racially biased language or other bias based on patients’ social identities. To our knowledge, no standardized handoff recommendations have done so.

### Limitations

Our study has several limitations. First, we simulated the handoff experience, so the influence of biased language could be different in clinical settings. Second, clinical information recall accuracy could have been negatively affected by the duration of the simulated handoffs, as biased-language handoffs were 16 seconds longer on average than neutral versions. This difference in duration, however, is much smaller than our prior recorded handoffs,^25^ where handoffs with any type of bias were 61 seconds longer than those without bias (162 vs 101 seconds; *P* < .001), and we controlled for handoff duration in adjusted models. Third, our study was conducted among residents and medical students at 2 academic medical centers; our findings might not generalize to clinicians in other practice settings. Fourth, we did not explicitly state the race of the hypothetical patients or measure the degree of racial bias or perceived patient race by participants, even though the original biased language all described Black patients.^25^ Fifth, the biased versions of the handoff were at times more colloquial than the neutral versions, which could affect clinical information recall separately from biased language, but recall was also lower when there was a biased takeaway.

## Conclusions

This survey study adds to the growing evidence about racially biased clinician communication. We found that residents and medical students who received handoffs about Black patients with biased language, compared with neutral language, retained the clinical information less accurately and had less positive attitudes, particularly empathy, about such patients. Our study’s demonstration of the impact of biased language on the clinical accuracy of handoff information indicates a need to address racially biased language through standardized handoff protocols as recommended by the Accreditation Council for Graduate Medical Education. While more research is needed to fully understand the impact of biased language on the care, health, and well-being of patients, the time has come to decrease racially biased language in clinician handoffs.

## Notes

Supplement 1. **eAppendix** A. Internal Medicine Handoff Survey **eAppendix** B. Pediatric Handoff Survey

Supplement 2.
Data Sharing Statement
