# Supplementary material for: Biased Language in Simulated Handoffs and Clinician Recall and Attitudes
Source: JAMA Netw Open. 2024 Dec 17;7(12):e2450172. doi: 10.1001/jamanetworkopen.2024.50172 (PMC11653120; doi:10.1001/jamanetworkopen.2024.50172)
Supplement: Supplement 1. — eAppendix A. Internal Medicine Handoff Survey eAppendix B. Pediatric Handoff Survey [file jamanetwopen-e2450172-s001.pdf]

Supplementary Online Content

Wesevich A, Langan E, Fridman I, Patel-Nguyen S, Peek ME, Parente V. Biased language in simulated handoffs and clinician recall and attitudes. *JAMA Netw Open*. 2024;7(12):e2450172. doi:10.1001/jamanetworkopen.2024.50172

eAppendix A. Internal Medicine Handoff Survey
eAppendix B. Pediatric Handoff Survey

This supplementary material has been provided by the authors to give readers additional information about their work.

© 2024 Wesevich A et al. *JAMA Network Open*.

eAppendix A. Internal Medicine Handoff Survey

Stereotype Handoff Block
Randomized to biased versus neutral, and order of handoff blocks also randomized. Participant listened to audio clip, but transcriptions are below.

Biased
This is a 74-year-old lady came in altered with pyuria. I am treating her for a UTI. She's on day two of three for her ceftriaxone. Her altered mental status is resolving. Her baseline now is A&Ox3 but a little bit irritable and like, skeptical. Like grouchy I guess is a good word for her. So just like, try not to go in that room if you don't have to. She's on, like I said, ceftriaxone. If she fevers again, I would get another urine culture and get blood cultures. No need to add vanc unless she's hemodynamically unstable. She'll probably, *hopefully* (!) go tomorrow. I'll put something in a PRN for her in case she gets agitated. She's naïve to all, like antipsychotics and everything, so no strong preference for her.

Neutral
This is a 74-year-old female with pyuria and altered mental status. I am treating her for a UTI. She's on day two of three for her ceftriaxone. Her mental status is improved to her baseline of A&Ox3. She has had a difficult admission with her changes in mental status. If she fevers again, I would get another urine culture and get blood cultures. No need to add vanc unless she's hemodynamically unstable. She'll probably go tomorrow. I'll put something in a PRN for her in case she gets agitated. She's naïve to all, like antipsychotics and everything, so no strong preference for her.

List three things you remember from this handover

1) ______________
2) ______________
3) ______________

What did the team recommend you do if this patient has a fever, but is otherwise hemodynamically stable, overnight?

A. Broaden coverage to vancomycin
B. Go assess the patient
C. Nothing if hemodynamically stable
D. Repeat blood and urine cultures

Not every patient and their family is regarded the same.

Compared to the average patient and family…

	Much less than average	Less than average	Average	More than average	Much more than average
How much do you like this patient and/or family?					

© 2024 Wesevich A et al. *JAMA Network Open*.

(liking means warmth and enthusiasm)					
How much empathy do you have for this patient and/or family?					
How much respect do you have for this patient and/or family?					

Thinking about this patient and their family, please indicate whether you tend to agree or disagree with the following statement

	Strongly Disagree	Disagree	Neither Agree nor Disagree	Agree	Strongly Agree
This patient and/or family is frustrating to take care of					
This patient and/or family makes me feel glad I went into medicine					

In your opinion, how likely is this patient and/or family to…

	Much less than average	Less than average	Average	More than average	Much more than average
Over-report (exaggerate) discomfort?					
Fail to comply with medical advice?					
Try to manipulate you or other providers?					
Abuse drugs, including alcohol?					

© 2024 Wesevich A et al. *JAMA Network Open*.

<u>Blame Handoff Block</u>
Randomized to biased versus neutral, and order of handoff blocks also randomized. Participant listened to audio clip, but transcriptions are below.

Biased
This is a 72-year-old female with hypertension, diabetes, and ovarian cancer. She is now cancer-free as of last month when she last saw her oncologist. She had been diagnosed with ovarian cancer about 2 years ago and at that time was like, “Well, I don’t have to worry about my sugars anymore.” And really stopped checking them. She takes Janumet at home but cut her dose in half two months ago because she was having some falls and thought she was getting hypoglycemic but never actually checked her blood glucose. She came in yesterday with a blood glucose in the 700s. She got fluids, potassium, and IV insulin so far. If her gap is closed, we can probably just keep her off an insulin drip, and it’ll be subcutaneous Lantus. Weight-based would be 20 units. You can consider, if you have other plans, but otherwise, and then in addition a sliding scale with regular and I would say just like a low. She’s currently NPO but if her sugars come down okay, if she’s like less than 300, you can let her eat if she’s bugging you.

Neutral
This is a 72-year-old female with hypertension, diabetes, and ovarian cancer coming in with hyperglycemia. So, she is now cancer-free as of last month when she last saw her oncologist. She’s been more focused on her ovarian cancer and stopped checking her glucose. She takes Janumet at home but cut her dose in half after experiencing hypoglycemic symptoms. She came in yesterday with a blood glucose in the 700s. She got fluids, potassium, and IV insulin so far. If her gap is closed, we can probably just keep her off an insulin drip, and it’ll be subcutaneous Lantus. Weight-based would be 20 units. You can consider, if you have other plans, but otherwise, and then in addition a sliding scale with regular and I would say just like a low. She’s currently NPO but if her sugars come down okay, if she’s like less than 300, you can remove her NPO status and order her a diet.

List three things you remember from this handover
1) _______________
2) _______________
3) _______________

What did the team recommend that you do if her glucose goes below 300?
A. Allow PO intake
B. Decrease sliding scale insulin dose
C. Stop IV fluids
D. Stop insulin drip

Not every patient and their family is regarded the same.

Compared to the average patient and family…

	Much less than average	Less than average	Average	More than average	Much more than average

© 2024 Wesevich A et al. *JAMA Network Open*.

How much do you like this patient and/or family? (liking means warmth and enthusiasm)					
How much empathy do you have for this patient and/or family?					
How much respect do you have for this patient and/or family?					

Thinking about this patient and their family, please indicate whether you tend to agree or disagree with the following statement

	Strongly Disagree	Disagree	Neither Agree nor Disagree	Agree	Strongly Agree
This patient and/or family is frustrating to take care of					
This patient and/or family makes me feel glad I went into medicine					

In your opinion, how likely is this patient and/or family to…

	Much less than average	Less than average	Average	More than average	Much more than average
Over-report (exaggerate) discomfort?					
Fail to comply with medical advice?					
Try to manipulate you or other providers?					
Abuse drugs, including alcohol?					

© 2024 Wesevich A et al. *JAMA Network Open*.

Doubt Handoff Block

Randomized to biased versus neutral, and order of handoff blocks also randomized. Participant listened to audio clip, but transcriptions are below.

Biased

This is a 62-year-old gentleman with prostate cancer for which he's on ART and also radiotherapy. He presented with like a month of nausea, vomiting, and diarrhea right after he started his radiation treatment. In the ED, it was found that he was like severely acidotic. 7.1. His bicarb was low, like 15. His potassium was 2.1. So, he had all these metabolic derangements and then on top of it he had a creatinine of 9.1. So, we‘ve been fluid resuscitating him with bicarb and checking his RFP and mag Q6. So that's what I'll need your help with tonight. His potassium is, the last one was like 2.7. We've been constantly repleting him, but he kind of is just chilling there. But his pH is slowly improving. So, at 11 and 5 AM, I think the orders are for 9 PM and 3 AM. So, if you could check on those and replete them if they need to be repleted.

The other thing for him, he is complaining of 10 out of 10 pain at like his bunions. Got bilateral bunions. I told the nurse to let the new nurse know not to page you about this. Because it's like, if he's looking away and you tap on it, it doesn't hurt. He's already got Tylenol and a lidocaine patch on it. Literally, don't do anything for that. Yeah, ha. It's a bunion. It's not gout. But the main thing for overnight is just repleting his electrolytes.

Neutral

This is a 62-year-old gentleman with prostate cancer for which he's on ART and also radiotherapy. He presented with like a month of nausea, vomiting, and diarrhea right after he started his radiation treatment. In the ED, it was found that he was like severely acidotic. 7.1. His bicarb was low, like 15. His potassium was 2.1. So, he had all these metabolic derangements and then on top of it he had a creatinine of 9.1. So, we‘ve been fluid resuscitating him with bicarb and checking his RFP and mag Q6. So that's what I'll need your help with tonight. His potassium is, the last one was like 2.7. We've been constantly repleting him, but he kind of is just chilling there. But his pH is slowly improving. So, at 11 and 5 AM, I think the orders are for 9 PM and 3 AM. So if you could check on those and replete them if they need to be repleted.

The other thing for him, he has bilateral bunions that are causing 10 out of 10 pain. I don't expect bunions to be that painful, and it doesn't look like gout. For now, I ordered Tylenol and a lidocaine patch for the pain and to see if that helps. But the main thing for overnight is just repleting his electrolytes.

List three things you remember from this handover

1) ______________
2) ______________
3) ______________

What lab value did the team ask you to follow-up and act upon overnight?

A. Creatinine
B. Magnesium

© 2024 Wesevich A et al. *JAMA Network Open*.

C. **Potassium**
D. Sodium

Not every patient and their family is regarded the same.

Compared to the average patient and family…

	Much less than average	Less than average	Average	More than average	Much more than average
How much do you like this patient and/or family? (liking means warmth and enthusiasm)					
How much empathy do you have for this patient and/or family?					
How much respect do you have for this patient and/or family?					

Thinking about this patient and their family, please indicate whether you tend to agree or disagree with the following statement

	Strongly Disagree	Disagree	Neither Agree nor Disagree	Agree	Strongly Agree
This patient and/or family is frustrating to take care of					
This patient and/or family makes me feel glad I went into medicine					

In your opinion, how likely is this patient and/or family to…

	Much less than average	Less than average	Average	More than average	Much more than average
Over-report (exaggerate) discomfort?					
Fail to comply with medical advice?					
Try to manipulate you or other providers?					
Abuse drugs, including alcohol?					

© 2024 Wesevich A et al. *JAMA Network Open*.

<u>Demographic Block</u>
Residents and medical students had slightly different versions as notated below.

Residents
What residency program are you in?
A. Medicine
B. Pediatrics
C. Medicine-Pediatrics
D. Other (example: Child Neurology)

At what institution do you practice?
A. Duke University
B. University of Chicago
C. Prefer not to answer

What is your PGY level?
A. 1
B. 2
C. 3
D. 4

Medical Students
What is your intended specialty?
A. Medicine
B. Pediatrics
C. Surgery
D. Other
E. Prefer not to answer

At what institution are you enrolled as a student?
A. Duke University
B. University of Chicago
C. Prefer not to answer

What is your year in medical school?
A. 1
B. 2
C. 3
D. 4
E. Prefer not to answer

Both Residents and Medical Students
What is your age in years? ______

What is your gender?
A. Female

© 2024 Wesevich A et al. *JAMA Network Open*.

B. Male
C. Non-Binary
D. Other
    a. Describe other gender (optional) ____
E. Prefer not to answer

Which racial and ethnic categories best describe you? Select all that apply.

- American Indian or Alaskan Native
- Black or African American
- East Asian (eg Chinese, Korean, Japanese)
- Hispanic or Latin American
- Middle Eastern or Northern African
- Native Hawaiian or Other Pacific Islander
- South Asian (eg Indian, Pakistani)
- South East Asian (eg Cambodian, Filipino, Vietnamese, Hmong)
- White
- Other race or ethnicity not listed here
    - (Optional) Please specify other racial or ethnic group: _______
- Prefer not to answer

© 2024 Wesevich A et al. *JAMA Network Open*.

eAppendix B. Pediatric Handoff Survey

Stereotype Handoff Block

Randomized to biased versus neutral, and order of handoff blocks also randomized. Participant listened to audio clip, but transcriptions are below.

Biased

This is a 6-month-old male, ex-23-weeker who was discharged from the NICU just last month and is here for concern for seizure-like activity. At home this morning with his mom and home health nurse, he had an episode for about 30 seconds where he was staring off into space. Mom waved his hand in front of him, and he didn't really seem to react. Then his head began to shake. But she describes it as kind of like a subtle tremor and not- and like his extremities, rest of his body, like everything else was fine. But it hasn't happened since then. And it's possible that he had another similar episode yesterday, but mom's not sure if she would characterize it as that. It's... just some general context, mom does seem a little bit frustrated about being in the hospital in general. Like literally screamed at me because she thought I was misgendering her baby when I was instead referring to the nurse. Mom's a little excitable. I would say like, she knows that he's going on EEG. I talked to her at length. Made sure I got all his feeds and all his meds exactly like she wanted them. So, hopefully you won't have a lot of discussion overnight, but just as an FYI, that's the situational awareness socially... And so, I apologize in advance if you get like a "We want to leave right now." Obviously, I don't anticipate it. We both talked to her about the necessity for the admission. And she didn't like say to me that she wanted to go. She just was in general frustrated. He's on monitoring. He's on the regular seizure pathway if he does have these episodes or like seizes. Neuro's obviously aware of him. Yeah, there shouldn't be anything for you guys to really do overnight.

Neutral

This is a 6-month-old male, born at 23 weeks who was discharged from the NICU just last month and is here for concern of seizure-like activity. At home this morning with his mom and home health nurse, he had an episode for about 30 seconds where he was staring off into space, not responding, and then his head began to shake. She describes it as kind of like a subtle tremor and not- and like his extremities, rest of his body, like everything else was fine. It's possible that he had a similar episode yesterday. Just some general context, mom is disappointed to be back in the hospital so soon after his discharge from the NICU and is wondering about the need to be back inpatient. I spent a while talking to her about the importance of capturing an event on EEG to know if these are seizures. I also went over his home meds and feeds with her to make sure they were correct and to avoid any errors. He's on monitoring. He's on the regular seizure pathway if he does have these episodes or like seizes. Neuro's obviously aware of him. Yeah, there shouldn't be anything for you guys to really do overnight.

List three things you remember from this handover

1) _______________
2) _______________
3) _______________

© 2024 Wesevich A et al. *JAMA Network Open*.

What was the description of the spell or seizure-like activity at home that prompted his admission?

A. Arm rhythmic jerking
B. Full-body tonic-clonic movements
C. Head shaking and tremor
D. Lip smacking

Not every patient and their family is regarded the same.

Compared to the average patient and family…

	Much less than average	Less than average	Average	More than average	Much more than average
How much do you like this patient and/or family? (liking means warmth and enthusiasm)					
How much empathy do you have for this patient and/or family?					
How much respect do you have for this patient and/or family?					

Thinking about this patient and their family, please indicate whether you tend to agree or disagree with the following statement

	Strongly Disagree	Disagree	Neither Agree nor Disagree	Agree	Strongly Agree
This patient and/or family is frustrating to take care of					
This patient and/or family makes me feel glad I went into medicine					

In your opinion, how likely is this patient and/or family to…

	Much less than average	Less than average	Average	More than average	Much more than average
Over-report (exaggerate) discomfort?					
Fail to comply with medical advice?					
Try to manipulate you or other providers?					

© 2024 Wesevich A et al. *JAMA Network Open*.

Blame Handoff Block

Randomized to biased versus neutral, and order of handoff blocks also randomized. Participant listened to audio clip, but transcriptions are below.

Biased

This is a 13-month-old with congenital hypotonia, developmental delay, and failure to thrive being admitted for acute weight loss and failure to thrive. She's lost like a pound and a half over the last month. She-let's see. So, it sounds like it's, she's been worked up outpatient for a lot of different metabolic and congenital things that have all been negative. So, we're thinking it's possibly part of her neurodevelopmental disorder. So, she's not like able to take in a good amount of food or eat well. And then the other part of it too—it sounds like she was doing formula at one point which, talking to mom just now, seems like they're not doing that anymore. We were told that mom was like hypo-diluting formula—but I guess when they told her that maybe she decided to interpret that as, "Oh, that's wrong. I just won't use formula then?" So, it could be that she's just not getting enough like nutritional, like enough in to meet her nutritional demand. She takes solid food, maybe half a handful a day, and some rice cereal diluted in almond milk. Mom also explicitly said like, "I'm afraid of overfeeding my baby." Which is shocking, given like she's first percentile growth. So, I think there's like some other stuff going on. I forgot to mention that part too. There's definitely something going on with mom. Her other labs that she's had so far have looked fine. I put her on the toddler 1- to 3-year-old diet. There's nothing really else for you guys to do. Tomorrow, big thing for her is just different consults.

Neutral

This is a 13-month-old with congenital hypotonia, developmental delay, and failure to thrive being admitted for acute weight loss and failure to thrive. She's lost like a pound and a half over the last month. She-let's see. So, it sounds like it's, she's been worked up outpatient for a lot of different metabolic and congenital things that have all been negative. So, we're thinking it's possibly part of her neurodevelopmental disorder. So, she's not like able to take in a good amount of food or eat well. It also sounds like she may not be getting enough nutritionally to meet her demands as there were some miscommunications about her formula mixing. She is no longer on formula, but gets rice cereal mixed with almond milk and a small amount of solids. Her other labs that she's had so far have looked fine. I put her on the toddler 1- to 3-year-old diet. There's nothing really else for you guys to do. Tomorrow, big thing for her is just different consults.

List three things you remember from this handover

1) _______________
2) _______________
3) _______________

What diet is this child ordered for in the hospital?

A. Fortified infant formula
B. Nasogastric tube feeds
C. Pediasure supplements
D. Toddler diet

© 2024 Wesevich A et al. *JAMA Network Open*.

Not every patient and their family is regarded the same.

Compared to the average patient and family…

	Much less than average	Less than average	Average	More than average	Much more than average
How much do you like this patient and/or family? (liking means warmth and enthusiasm)					
How much empathy do you have for this patient and/or family?					
How much respect do you have for this patient and/or family?					

Thinking about this patient and their family, please indicate whether you tend to agree or disagree with the following statement

	Strongly Disagree	Disagree	Neither Agree nor Disagree	Agree	Strongly Agree
This patient and/or family is frustrating to take care of					
This patient and/or family makes me feel glad I went into medicine					

In your opinion, how likely is this patient and/or family to…

	Much less than average	Less than average	Average	More than average	Much more than average
Over-report (exaggerate) discomfort?					
Fail to comply with medical advice?					
Try to manipulate you or other providers?					

© 2024 Wesevich A et al. *JAMA Network Open*.

Doubt Handoff Block

Randomized to biased versus neutral, and order of handoff blocks also randomized. Participant listened to audio clip, but transcriptions are below.

Biased

This is a 14-year-old with optic neuritis getting IVIg today. That's what the intern was getting paged about. She just did the second decrease in IVIg infusion rate since he was complaining of tightness in his chest, and so she is going over to assess whether there's concern for anaphylaxis versus not. Part of the reason why I have a concern for possibly not is he, at baseline, I don't know if you have met him, is a pretty darn anxious kid. She's gonna go look at him. That being said, we will treat with either Benadryl or epi plus Benadryl depending on what's going on there.

Neutral

This is a 14-year-old with optic neuritis getting IVIg today. That's what the intern was getting paged about. She just did the second decrease in IVIg infusion rate since he was complaining of tightness in his chest, and so she's going over to assess for anaphylaxis. We will treat with either Benadryl or epi plus Benadryl depending on what's going on there.

List three things you remember from this handover

1) ______________
2) ______________
3) ______________

What change had the day intern already done for his chest tightness?

A. Albuterol nebulizer trial
B. Benadryl dose
C. Epinephrine injection
D. Slowed down infusion rate

Not every patient and their family is regarded the same.

Compared to the average patient and family…

	Much less than average	Less than average	Average	More than average	Much more than average
How much do you like this patient and/or family? (liking means warmth and enthusiasm)					
How much empathy do you have for this patient and/or family?					
How much respect do you have for this patient and/or family?					

© 2024 Wesevich A et al. *JAMA Network Open*.

Thinking about this patient and their family, please indicate whether you tend to agree or disagree with the following statement

	Strongly Disagree	Disagree	Neither Agree nor Disagree	Agree	Strongly Agree
This patient and/or family is frustrating to take care of					
This patient and/or family makes me feel glad I went into medicine					

In your opinion, how likely is this patient and/or family to…

	Much less than average	Less than average	Average	More than average	Much more than average
Over-report (exaggerate) discomfort?					
Fail to comply with medical advice?					
Try to manipulate you or other providers?					

© 2024 Wesevich A et al. *JAMA Network Open*.

Demographic Block

Residents and medical students had slightly different versions as notated below.

Residents

What residency program are you in?

A. Medicine
B. Pediatrics
C. Medicine-Pediatrics
D. Other (example: Child Neurology)

At what institution do you practice?

A. Duke University
B. University of Chicago
C. Prefer not to answer

What is your PGY level?

A. 1
B. 2
C. 3
D. 4

Medical Students

What is your intended specialty?

A. Medicine
B. Pediatrics
C. Surgery
D. Other
E. Prefer not to answer

At what institution are you enrolled as a student?

A. Duke University
B. University of Chicago
C. Prefer not to answer

What is your year in medical school?

A. 1
B. 2
C. 3
D. 4
E. Prefer not to answer

Both Residents and Medical Students

What is your age in years? ______

What is your gender?

A. Female

© 2024 Wesevich A et al. *JAMA Network Open*.

B. Male
C. Non-Binary
D. Other
    a. Describe other gender (optional) ____
E. Prefer not to answer

Which racial and ethnic categories best describe you? Select all that apply.

- American Indian or Alaskan Native
- Black or African American
- East Asian (eg Chinese, Korean, Japanese)
- Hispanic or Latin American
- Middle Eastern or Northern African
- Native Hawaiian or Other Pacific Islander
- South Asian (eg Indian, Pakistani)
- South East Asian (eg Cambodian, Filipino, Vietnamese, Hmong)
- White
- Other race or ethnicity not listed here
    - (Optional) Please specify other racial or ethnic group: _______
- Prefer not to answer

© 2024 Wesevich A et al. *JAMA Network Open*.
