# Supplementary material for: Biased Language in Simulated Handoffs and Clinician Recall and Attitudes
Source: JAMA Netw Open. 2024 Dec 17;7(12):e2450172. doi: 10.1001/jamanetworkopen.2024.50172 (PMC11653120; doi:10.1001/jamanetworkopen.2024.50172)
Supplement: Supplement 2. — Data Sharing Statement [file jamanetwopen-e2450172-s002.pdf]

Data Sharing Statement

Wesevich. Biased Language in Simulated Handoffs and Information Recall and Attitudes Toward Patients. *JAMA Netw Open*. Published December 17, 2024.
doi:10.1001/jamanetworkopen.2024.50172

Data
Data available: No

Additional Information
Explanation for why data not available: Sharing individual-level responses from residents and medical students will be considered on a case-by-case basis given the privacy of the trainees who participated.
